# Investigation on the Toxicity of Nanoparticle Mixture in Rainbow Trout Juveniles

**DOI:** 10.3390/nano13020311

**Published:** 2023-01-11

**Authors:** Joelle Auclair, Patrice Turcotte, Christian Gagnon, Caroline Peyrot, Kevin James Wilkinson, François Gagné

**Affiliations:** Aquatic Contaminants Research Division, Environment and Climate Change Canada, 105 McGill, Montréal, QC H2Y 2E7, Canada

**Keywords:** nanoparticle, mixture, oxidative stress, protein damage, genotoxicity, acetylcholinesterase, rainbow trout

## Abstract

The environmental impacts of nanoparticle mixtures in the aquatic environment is not well understood. The purpose of this study examined the sub-lethal toxicity of low concentrations (ug/L range) of selected nanoparticles alone and in mixtures in juvenile trout. Fish were exposed to to individual and two environmentally relevant mixtures of silver (nAg), copper oxide (nCuO) and cerium oxide (nCeO) nanoparticles for 96 h at 15 °C. After the exposure period, fish were depurated overnight and tissue levels in Ag, Ce, Cu and Zn were determined along with a suite of effects biomarkers such as oxidative stress/inflammation, denatured protein tagging (ubiquitin), DNA strand breaks (genotoxicity) and acetylcholinesterase (AChE) activity. The data showed that these nanoparticles behaved as suspended matter but were nevertheless bioavailable for fish with bioconcentration factors of 6, 8 and 2 for nAg, nCeO and nCuO respectively. Only nCuO alone increased malonaldehyde (lipid peroxidation) contents but all nanoparticles increased DNA damage, protein-ubiquitin labeling, and decreased AChE activity. Globally, the toxicity of nCeO and nCuO was generally stronger than nAg, and antagonist effects were found in the mixtures. The interactions involved in these antagonisms are not well understood but do not involve the liberation of free ions and labile zinc in tissues. In conclusion, the bioavailability and toxicity of these nanoparticles are influenced by mixtures of nanoparticles, which is likely to occur in contaminated environments.

## 1. Introduction

Nanoparticles are inadvertently released into the environment from the nanotechnology industry. Indeed, nanotechnology products pervade many sectors of our economy such as micro(nano)electronics, bacteria-resistant medical and personnel devices, odor control, fuel additives, and various materials (ceramics, paints). Inorganic nanoparticles comprise reduced elements and oxides such as silver (nAg), copper (nCu or nCuO), cerium (nCeO_2_), and zinc (nZnO), which are often detected in municipal wastewaters and in the vicinity of largely populated and industrialized area [1,2,3]. These nanoparticles find their way into receiving aquatic habitats from municipal treated and untreated wastewaters, road (tire) erosion dusts [4], and atmospheric deposition (exhaust). Hence, organisms are likely exposed to a cocktail or mixture of nanoparticles in addition to dissolved contaminants in the environment. In addition, global warming increases precipitation events leading to rainfall overflows where untreated wastewaters combine with rain to be diverted directly into the water bodies given the limited capacity of many sewage wastewaters works. For example, nAg and nCuO are embeded in clothing to prevent foul odors from microorganisms and nZnO are used as sunscreens in cosmetics and sun protection creams are commonly found in municipal effluents [5]. Cerium-based nanoparticles are often found as stabilizers in fuel additives and ceramic paints and electronic devices [6]. Although not very soluble, they are found in a suspended matter as fine particles and sediments. The antimicrobial effects of nCuO in addition to nAg are also used for cosmetics, clothes, and recently as an additive in chirurgical masks during the COVID-19 pandemic and other air-borne diseases [7]. Unfortunately, these masks are carelessly disposed of in streets finding their way in sewers, if not in solid waste deposal sites, thus contributing to the release of nCuO and nanoplastic polymers in aquatic ecosystems.

The toxicity of nanoparticles first focused on the intrinsic toxicity of individual nanoparticles and often compared with the release of its ionic counterpart (e.g., nAg and Ag^+^). Studies dealing with mixtures of aquatic organisms are only in the beginning [8,9]. Indeed, nanoparticles could interact with each other, sometimes through the release of ions, with other contaminants (act as vectors sometimes coined as Trojan horse effects) and natural compounds such as organic/inorganic matter found in both the dissolved and suspended compartments. Hence, all kinds of interactions could be observed such as synergy between contaminants such as bisphenol A and titanium dioxide nanoparticles [10], different geometries of nanoparticles [11,12], antagonisms between different types of nanoparticles [13] and interactions with natural organic matter [14]. All these variables can interact in the environment making the prediction of toxic outcomes difficult for the risk assessment community. Moreover, the toxicity of nanomaterials results not only from the liberation of ions or monomers but from the size, form, surface reactivity, and binding of small contaminants (metal and organic vectors). In general, nanoparticles are generally less toxic than the elemental/molecular units but they can still produce long-term damage such as oxidative stress (lipid peroxidation), DNA damage, and altered protein conformation/denaturation [13,15,16]. The toxicity of nCuO was circa six times lower than for Cu(II) in rainbow trout [17] and the 5th percentile of the acute toxicity distribution was 10 and 150 µg/L for Cu(II) and nCuO respectively [18]. Nanomaterials could also introduce biophysical strains at the molecular level from the mere size and form of the nanoparticles such as the formation of liquid crystals in cells, altered enzyme activity by molecular crowding, and altered lipid metabolism [13,19]. In nCuO and nZnO mixtures, the toxicity of nCuO in the presence of a non-toxic concentration of nZnO was increased to an LC50 of 0.1 mg/L compared to nCuO alone with an LC50 > 1 mg/L [20]. One of the reasons why toxicity is lower for some metallic nanoparticles, is that metal loadings could be reduced by other nanoparticles. Hence, the objective of this study was to examine the bioavailability and toxicity of three commonly-found nanoparticles in the environment in fish juveniles.

The purpose of this study was therefore to determine the bioavailability and toxicity of three commonly found elemental nanoparticles (nAg, nCeO, and nCuO) individually, and in two mixtures at environmentally relevant concentrations to in rainbow trout juveniles *Oncorhynchus mykiss*. The null hypothesis consists that the toxicity of single nanoparticles is the same in mixtures. Young of the year trout were exposed individually to nAg, nCeO, and nCuO and to two mixtures for 96 h at 15 °C following a standardized procedure. The fish were then analyzed for tissue contents in Ag, Ce, Cu, and Zn followed with a biomarker test battery related to oxidative stress, zinc homeostasis, genotoxicity and protein turnover. An attempt was made to identify mixture interactions such as additivity, synergy, or antagonisms.

## 2. Materials and Methods

### 2.1. Nanomaterial Characterization and Fish Exposure

The toxicity of the following nanoparticles (nAg, nCeO_2_ and nCuO) was determined individually and in 2 mixtures to *Oncorhynchus mykiss* trout juveniles (2–4 cm length). The fish were exposed to sublethal concentrations of 50 µg/L of nAg, 50 µg/L nCuO and 10 µg/L nCeO based on previous studies to ensure absence of acutely lethal effects [21,22]. Moreover, the selection of the 50 µg/L and 10 µg/L concentration was selected based on the observation that these elements are found in municipal effluents and combined sewer overflows at concentrations between 0.1 and 100 µg/L [23,24]. Mixture 1 consisted of 20 µg/L nAg, 20 µg/L nCuO and 5 µg/L nCeO (total elements of 45 µg/L) and mixture 2 consisted of the same initial concentrations (50, 50 and 10 µg/L; total 110 µg/L). A number of 10 fish was placed in 20 L aquariums for 24 h prior to exposure to the following 6 treatment groups: controls, nAg, nCeO, nCuO, mixture 1 and mixture 2. Controls consisted of aquarium water from tap water with the following physico-chemical characteristics following UV-treated and dechlorination (charcoal filter): conductivity: 250 µS × m^−1^, pH = 7.8, organic carbon content: 5 mg/L and total suspended solids <1 mg/L). The nanoparticles were obtained as water suspension to minimize hazards from the manipulation of fine dust. Cerium dioxide (nCeO_2_) nanoparticles were obtained from Sigma-Aldrich (Oakville, ON, Canada). Citrate-coated silver nanospheres (nAg) were purchased from nanoComposix Inc. (San Diego, CA, USA). A stock of copper oxide (nCuO) nanoparticles was obtained from US Research Nanomaterials (Houston, TX, USA). The nanoparticles were diluted first to 1 mg/L in MilliQ water and then to the given concentrations in the aquarium water to minimize aggregation during handling. The nanoparticle size distribution and Zeta potential were checked at the 1 mg/L concentration using dynamic light scattering instrument (Mobius Instrument with a laser at 532 nm; Wyatt Technologies, Santa Barbara, CA, USA). The instrument was calibrated with latex nanoparticle suspensions (Polyscience, Niles, IL, USA). Water samples (without added fish) were collected after 1 h and 48 h for total element assessments (Ag, Cu, Ce) by mass spectrometry as described below. At the end of the exposure period, the fish (N = 10) were kept in clean water overnight, and separated into 2 subgroups for elemental analysis (N = 3) and for biomarkers (N = 7) before freezing at −85 °C. For the biomarker group, the fish were anesthetized in 10 mg/L tricaine following the recommendation of the animal care committee, weighted and length determined for the condition factor (CF: weight/length) before storing at −85 °C.

### 2.2. Elemental Analysis in Water and in Fish

The exposure media (1 and 48 h) and fish were analyzed for Ag, Cu, Ce, and Zn using high-resolution plasma mass spectrometry (XSERIES 2 ICP-MS, Thermofisher Scientific, Nepean, MA, USA). For aquarium water, the dissolved fraction was analyzed following filtration of the water samples on a 0.45 µm cellulose acetate membrane filter and acidified to 1% nitric acid (Seastar Grade, Sydney, BC, Canada). For fish, each individual was grinded and mixed with one volume of HNO_3_ (16 N), HCl (12 N) and 30% H_2_O_2_ and heat digested in microwave vessels for 2 h. The samples were diluted to 12 mL with MilliQ water before analysis. The total elemental composition was determined using standard solutions of Ag, Ce, Zn and Cu chloride salts in 1% *v*/*v* nitric acid.

### 2.3. Biomarker Assessments

Fish were thawed on ice, the gills and livers removed and weighed for the hepatic or gill somatic index (gill or liver weights/fish weight) determinations. The still frozen tissues were homogenized in 5 volumes of 25 mM Hepes-NaOH, pH 7.4, containing 140 mM NaCl, 0.1 mM dithiothreitol and 1 µg/mL apoprotinin B. A hand-held Polytron tissue grinder was used to homogenize the sample at 4 °C and a portion of the homogenate was centrifuged at 15,000× *g* for 30 min at 2 °C to obtain the supernatant (S15 fraction). Total protein contents were determined using the protein-dye binding principle [25]. Calibration was achieved using serum bovine albumin. Lipid peroxidation was determined in the homogenates using a miniaturized version of the thiobarbituric acid reactants (TBARS) methodology [26]. The measurement of TBARS was performed by fluorescence at 540 nm excitation and 600 nm emission. The data were expressed as TBARS/mg proteins. Genotoxicity was determined using the fluorometric alkaline DNA precipitation assay [27,28,29]. DNA strand breaks remaining in the solution were determined using the hoescht dye and fluorescence at 360 nm excitation and 450 nm emission (Synergy-4, Microplate reader, Biotek, Winooski, VT, USA). Salmon sperm DNA was used for calibration and expressed as ug DNA/mg proteins. The levels of labile zinc (Zn) were also determined by the fluorescence probe methodology [30]. The S15 fraction was mixed with 5 volumes of 100 µM TSQ (6-methoxy-8-*p*-toluenesulfonamido-quinoline) probe in 20% DMSO in phosphate-buffered saline, pH 7.4. Fluorescence measurements were taken at 360 nm excitation and 460 nm emission (Synergy-4, Biotek Instruments, Winooski, VT, USA) using solutions of ZnSO_4_ for calibration. Data were expressed as relative fluorescence units (RFU)/mg proteins.

The enzyme activities of inflammation and neural activity were determined by arachidonate-dependent cyclooxygenase (aCOX) and acetylcholinesterase (AChE) respectively. They were also determined using spectrometric procedures as previously described [13]. The assays were performed in the S15 fraction in the presence of either 20 µM of arachidonic acid/10 µM of dichlorofluorescein (excitation at 485 nm and emission at 520 nm) or 0.5 mM acetylthiocholine/0.2 M dithionitrobenzoate (absorbance 412 nm) substrate reagents respectively. The data were expressed as RFU for fluorescein/min/mg proteins (aCOX) and increased absorbance at 412 nm/min/mg proteins (AChE). Finally, polyubiquitin protein staining was determined by enzyme-linked immunosorbent assay (ELISA) in the S15 fraction as previously described [31]. Briefly, a direct immunoassay was used with ubiquitin lys48-specific as the primary antibody (1/2000 dilution in PBS-albumin 0.5%; clone Apu2; EMD Millipore, Billerica, MA, USA) on S15 pretreated microplates (Immulon-4) at 1 ug/mL per well. The secondary antibody consisted of anti-rabbit IgG-peroxidase conjugate (1/5000 dilution in PBS/0.5% albumin; ADI-SAB-300, Enzo, NY, USA). Calibration was achieved with poly-ubiquitin complex (UB2-, K48-linked, Enzo Life Sciences, NY, USA) coated in three separate wells as with the blank (PBS only). Finally, peroxidase activity was determined using a highly sensitive chemiluminescence detection kit (BM Chemiluminescence ELISA substrate, Roche Diagnostics, Laval, QC, Canada). The data were expressed as ng of polyubiquitin/mg proteins.

## 3. Data Analysis

The exposure experiments were performed with N = 10 trouts per vessel and two replications of each treatment were used for each of the six treatment groups: controls, nAg, nCeO, nCuO, Mix 1 and Mix 2. A number of three fish were selected for bioaccumulation and N = 7 trouts for biomarkers analyses in each treatment group. Data normality was confirmed using the Shapiro-Wilks test and the data were subjected to covariance analysis with fish size (CF) as the covariable to control for size effects and Least Square Difference test was used to determine the difference between controls, individual nanoparticles and their mixtures (mix 1 and 2). Correlation and discriminant function analyses were performed to seek interrelationships between the effects biomarkers and similarities between the nanoparticles alone and the mixture (mix 1 and 2 were combined into one group). Significance was set at *p* < 0.05. All statistical analyses were performed with the SysStat software package (v 13.2, San Jose, CA, USA).

## 4. Results

**Figure 1:**
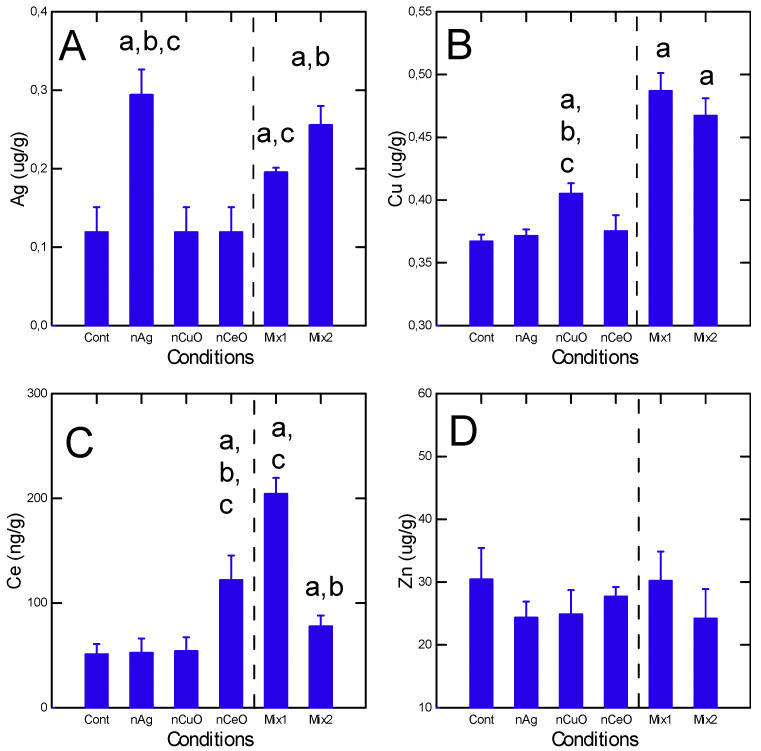
Metal uptake in fish exposed to nanoparticles and mixtures. The data represent the mean with the standard error for Ag (**A**), Cu (**B**), Ce (**C**) and Zn (**D**). The Mix1 consists of 20 µg/L of nAg and nCuO and 5 µg/L of nCeO giving a net concentral of 45 µg/L. The Mix2 consists of 50 µg/L of nAg and nCuO and 10 µg/L nCeO giving a net concentration of 110 µg/L. The letter a indicates significance relative to controls, b significance relative to Mix1, and c relative to Mix2.

In this study, juvenile fish were exposed to either 50 µg/L nAg or nCu, and 10 ug/L nCe alone and to 2 mixtures: mixture 1 (20 µg/L for nAg/nCu and 5 µg/L for nCe for a total of 45 µg/L) and mixture 2 (50 µg/L nAg/nCuO and10 µg/L nCe = 110 µg/L total). The dissolved concentrations of these elements (Ag, Cu Ce) were determined in the aquarium water after 1 h and 48 h to gain some information on the stability of nanoparticles alone and in mixtures over time (Table 1). For 50 µg/L nAg, the measured dissolved Ag was 48 after 1h and dropped to 33 µg/L (circa 70% of the added concentration) after 48 h. The levels of Ag were the same in both mixtures (taking into account that 20 µg/L nAg was added in mixture 1) suggesting that the nAg remained stable in the presence of nCeO and nCuO. For nCuO, about 8% of the added concentrations remained in solution after 1 h and remained stable at 96 h suggesting that nCuO formed aggregates (retained by the 0.45 µm pore filter). When added to the mixture, the measured Cu concentration remained the same in the nCuO group but dropped to 5% in mixtures 1 and 2. In the case of nCeO, about 12% of added nCeO remained in the dissolved phase (<0.45 µm) after 1h and was reduced by 7.5% after 96 h when singly added in water. In the mixtures, the proportion of Ce remaining in the solution was 7 and 8% for both mixtures 1 and 2 respectively after 1 h. At 96 h, the proportion of Ce dropped at 0.6 and 2% for mixtures 1 and 2 respectively. This suggests that nCuO and nCeO are found in the suspended matter compartment. The metal/element uptake of these nanoparticles was examined in fish tissues (Figure 1A–C). For nAg, tissue levels of Ag were significantly higher in fish exposed to nAg and mixtures 1 and 2. Total Ag in fish was lower when added to the mixture compared to nAg alone and control groups suggesting antagonism. For nCuO, tissue Cu levels were significantly higher than controls from both the nCuO alone and mixtures groups. The uptake of Cu was enhanced (1.2-fold for mixture 2) when added to the mixtures compared to the nCuO alone group. For nCeO, tissue levels of Ce were significantly higher in both the nCe alone and the mixtures groups. Tissue Ce levels were the highest in mixture 1 (exposed to 20 µg/L Ce as nCeO) and were significantly lower for mixture 2, which contained the same amount of Ce as the nCeO alone group. 

The morphological characteristics of rainbow trout were determined (Table 2). The CF (fish weight/length) remained unchanged between the 5 treatments (nAg, nCuO, nCeO, mixture 1, and mixture 2) and control groups. The HSI was significantly increased by the nCuO, nCeO, and the 2 mixtures compared to controls. The HSI from the single nAg group was significantly lower than the 2 mixtures group. Correlation analysis revealed that HSI was significantly correlated with tissue Cu (r = 0.45) and Ce (r = 0.47) levels. For the gill index, a decrease in the gill mass was observed in fish exposed to nCuO, nCeO, mixtures 1 and 2 groups. The gill index (gill weight/fish weight) was significantly correlated with Cu (r = −0.46) and Ce (r = −0.34) tissue levels in fish.

**Figure 2:**
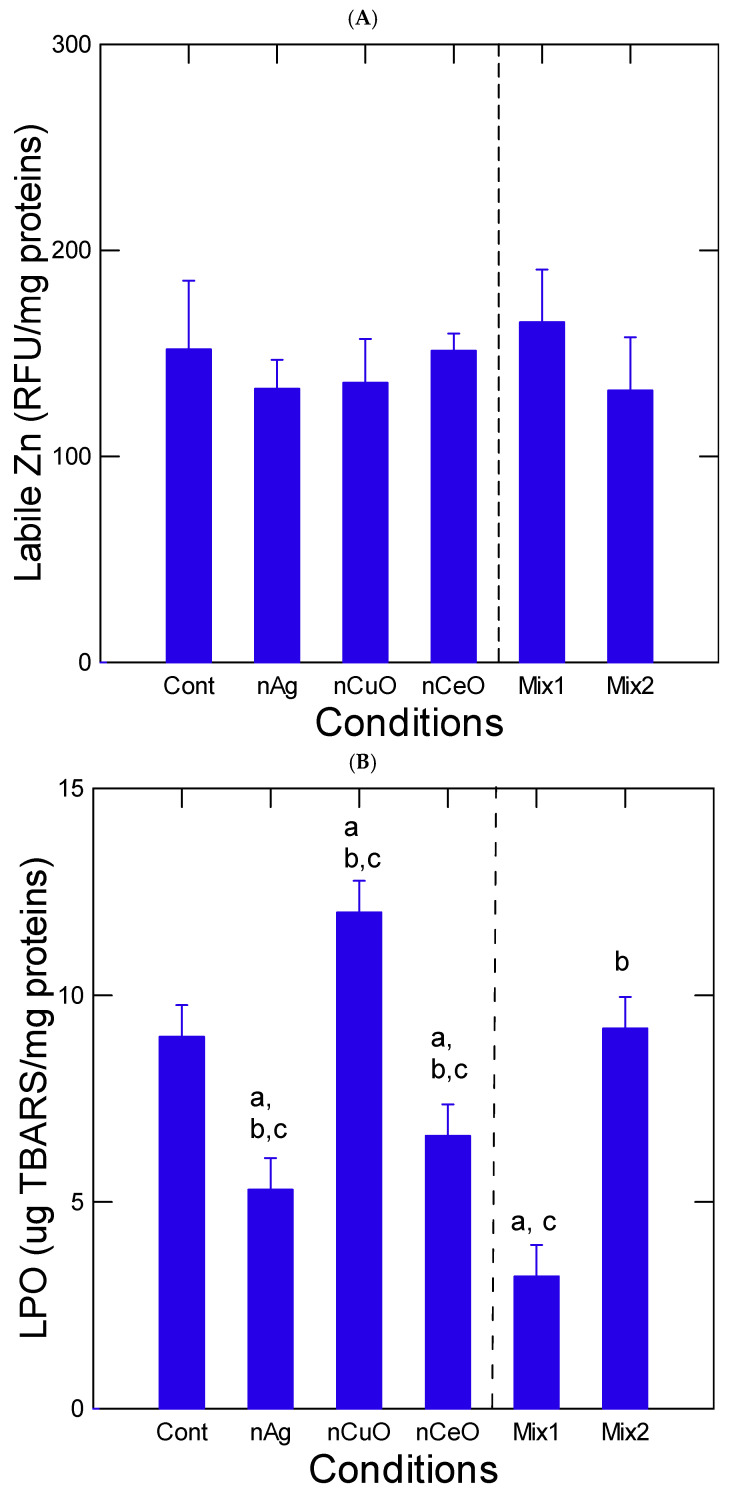

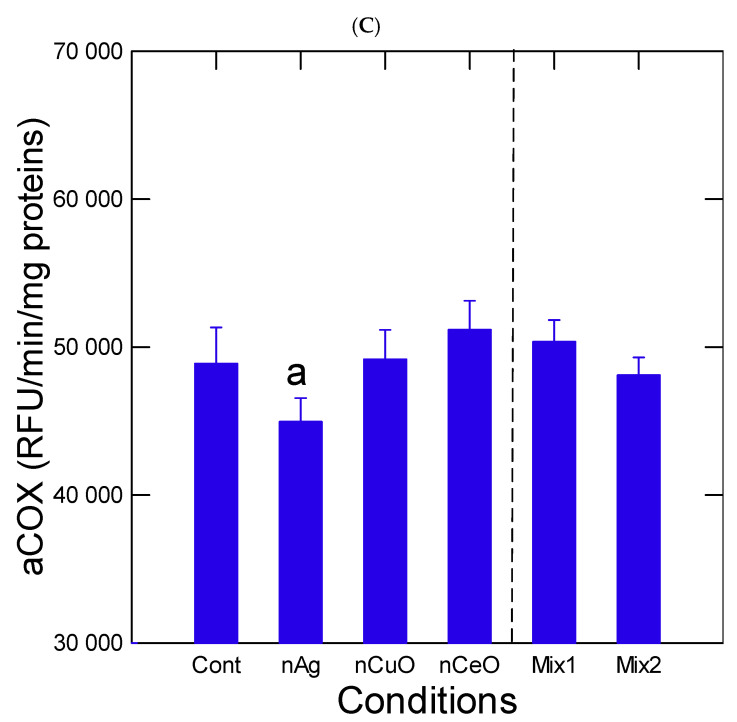
Oxidative stress in the liver of trout exposed to the nanoparticles. Oxidative stress was determined by labile Zn (**A**), LPO (**B**) and aCOX (**C**). The data represent the mean with the standard error. The letter a indicates significance relative to controls, b significance relative to Mix1 and c relative to Mix2.

The levels of oxidative stress were determined in the liver of juvenile trouts by measuring labile Zn, LPO, and aCOX (Figure 2). Tissue levels in labile Zn did not change between the treatment groups. Correlation analysis revealed that labile Zn was significantly correlated with total Zn (r = 0.95) in fish tissues. The LPO levels were increased in fish exposed to nCuO but decreased in fish exposed to nAg, nCeO and mixture 1 relative to controls. This could indicate that oxidative stress occurred at different times during the 96 h exposure period. The marker of inflammation, aCOX, was marginally decreased in the nAg alone group with no apparent interaction in the mixtures. LPO levels were significantly correlated with HSI (r = −0.46) and tissue Cu levels (r = −0.66). No correlations were found with aCOX activity. 

**Figure 3:**
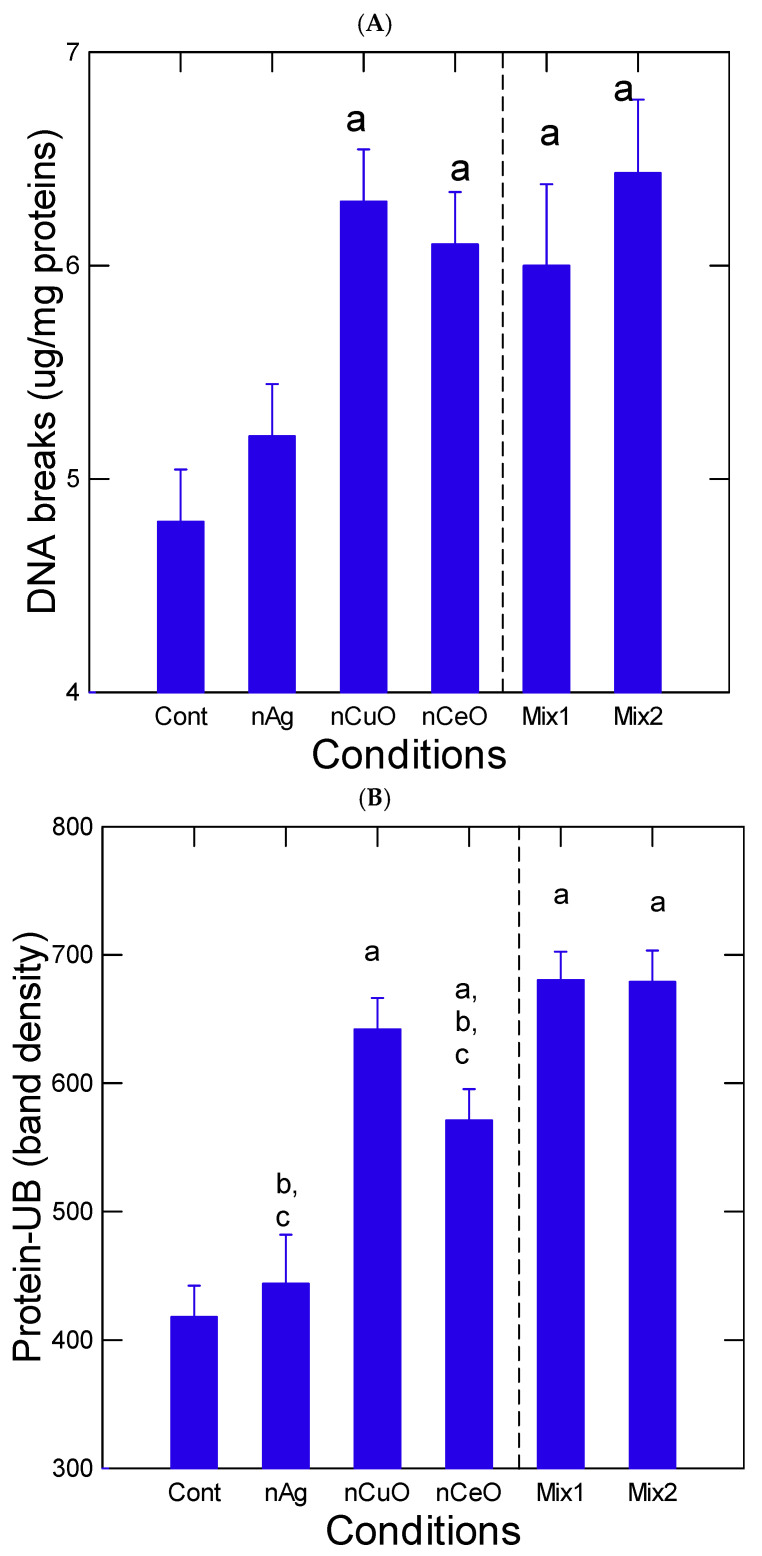
Hepatic DNA and protein damage in trout exposed to the nanoparticles. The data represent the mean with the standard error for DNA damage (**A**) and protein-ubiquitin tagging (**B**). The Mix1 consists of 20 µg/L of nAg and nCuO and 5 µg/L of nCeO giving a net concentral of 37 µg/L. The Mix2 consists of 50 µg/L of nAg and nCuO and 10 µg/L nCeO giving a net concentration of 110 µg/L. The letter a indicates significance relative to controls, b significance relative to Mix1, and c relative to Mix2.

**Figure 4:**
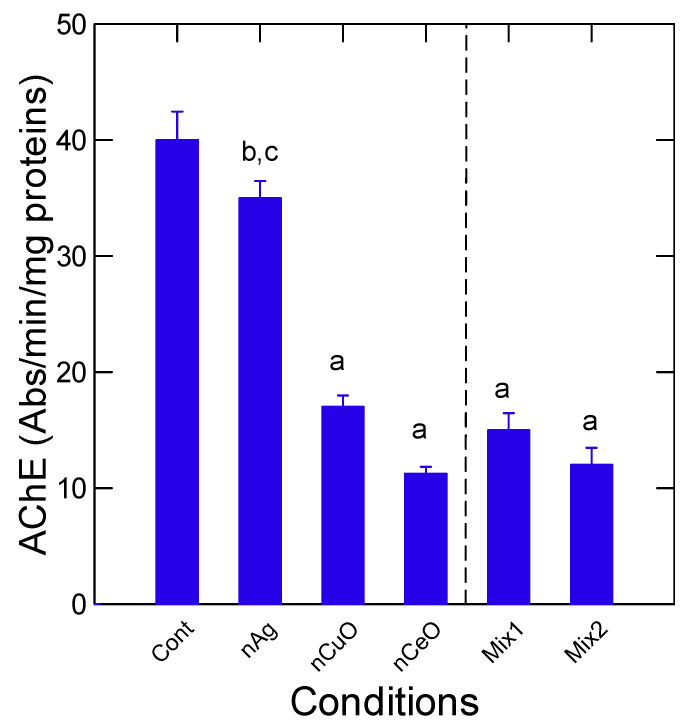
Hepatic acetylcholinesterase activity in trout exposed to nanoparticles. The data represent the mean with the standard error. The Mix1 consists of 20 µg/L of nAg and nCuO and 5 µg/L of nCeO giving a net concentration of 45 µg/L. The Mix2 consists of 50 µg/L of nAg and nCuO and 10 µg/L nCeO giving a net concentration of 110 µg/L. The letter a indicates significance relative to controls, b significance relative to Mix1, and c relative to Mix2.

Hepatic DNA and protein damage were determined in fish livers (Figure 3). Both DNA and protein damages were increased in fish exposed to nCuO, nCeO and to both mixtures relative to controls. Protein damage, as determined by polyubiquitin protein labeling, was also significantly increased by both mixtures compared to nCeO but not with nCuO highlighting different mixture effects. Correlation analysis revealed that DNA damage was significantly correlated with aCOX activity (r = 0.38). Protein-UB levels were significantly correlated with the gill index (r = −0.37) and tissue Cu levels (r = 0.46). The activity in AChE was measured in the liver as a general marker of neuroactivity (Figure 4). AChE activity was significantly reduced in fish exposed to nCuO, nCeO, and in both mixtures but not with nAg. Correlation analysis revealed that AChE was significantly correlated with HSI (r = 0.47) and tissue levels of Ce (r = 0.46).

**Figure 5:**
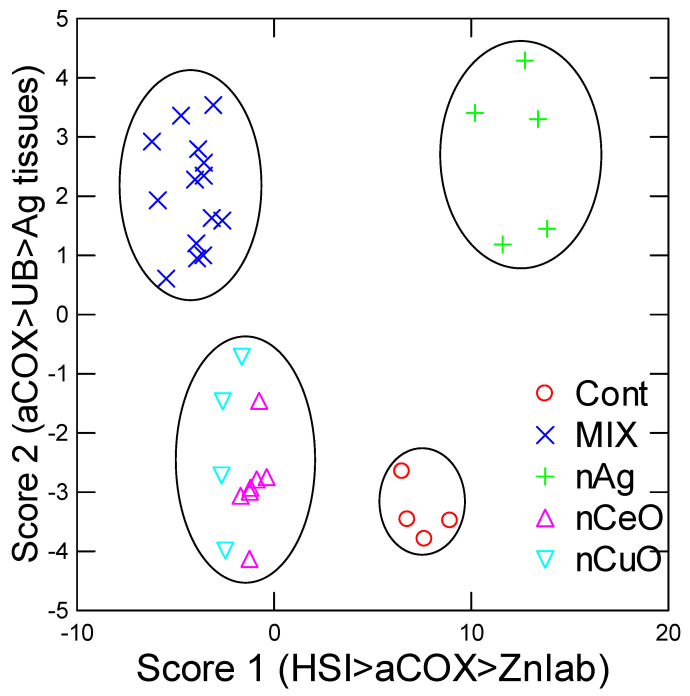
Discriminant function analysis of fish responses to nanoparticles. Discriminant function analysis was performed and revealed a mean classification efficiency of 71% and a total explained variance of 95%. The most important biomarkers are included in the parenthesis of each ax.

In an attempt to gain a more comprehensive view on the effects of individual nanoparticles and their mixtures in juvenile rainbow trout, a discriminant function analysis was performed (Figure 5). The analysis revealed that the treatment groups were correctly classified with a mean of 71% and 95% of the total variance was explained by the first two component scores. The mixtures 1 and 2 were combined into one mixture group during this analysis and yielded a distinct cluster from the other treatment groups. The control and nAg groups formed distinct clusters while the oxides (nCeO and nCuO) formed one cluster, which was closer to the mixture effects. Factorial analysis revealed that the following biomarkers explained most of the variance: HSI, aCOX, labile Zn, protein-UB, and Ag tissue levels. Based on these results, exposure to nanoparticles single and in mixtures leads to liver enlargement, oxidative stress, and protein damage.

## 5. Discussion

Based on the dissolved fraction of the nanoparticles in freshwater, nAg remained dissolved in greater proportions (70–96%) than the oxides (nCeO) and nCuO), which only 7–10% of the nanoparticle oxides remained in the dissolved phases. This was also found in a previous study where about 12% of added nCeO (16 nm diameter) remained in the dissolved fraction in aquarium water [21]. In another study, the proportion of nAg remaining in the dissolved fraction was in the order of 50% [32]. The occurrence of nCuO as aggregate suspensions was also found by others [33] suggesting that this nanoparticle is found as suspended matter in freshwater. This suggests that the route of exposure differed between nAg (dissolved) and oxide nanoparticles (suspended matter). Notwithstanding this, significant accumulation was found in fish after 12 h depuration (Figure 1). The ratio between tissue and water concentrations (a bioconcentration factor) was calculated giving a factor of 6, 8 and 2 for nAg, nCuO and nCeO respectively. This is consistent with the poor solubility of nCeO compared to nAg and nCuO. Increased levels of Ag and CuO could results from the liberation of the Ag and Cu ions compared to Ce either in the exposure media or in tissues. However, no significant increase in labile Cu^2+^ was found in tissues of mussels exposed to nCuO [34].

It is noteworthy that exposure of trout juveniles revealed increased oxidative stress (LPO) for nCuO only while nAg and nCeO did not show oxidative stress. The significant decrease in LPO for nAg and nCeO could indicate that oxidative stress occurred early during exposure where the observed response after 96 represents an over-compensation mechanism. The capacity of nAg to induce oxidative stress in fish is well known [15,35]. Cerium oxide nanoparticles were also shown to produce reactive oxygen species at toxic levels in various organisms because of Ce(III)/Ce(IV) redox couple [36]. The production of oxidative stress leads to LPO, lipofuscin accumulation (age-related pigments), and reduced life span in nematodes. However, nCeO was shown to protect against amine-coated nAg in *Labeo rohita* carp [37]. Indeed, nCeO was shown to scavenge oxygen radicals generated by nAg-induced oxidative stress. The Ce(III)/Ce(IV) redox couple forming at the surface of the nanoparticles mimics the activity of superoxide dismutase, peroxidase, and catalase involved in the inactivation of reactive oxygen species O_2_* (superoxide radical), OH*, and H_2_O_2_ [38]. Accumulation of yellow pigments in hepatic tissue was also seen due to necrosis of affected cells in fish perhaps from oxidative breakdown products related to age-related pigments.

The increase in DNA and protein damage with decreased AChE were the physiological biomarkers strongly affected by nanoparticles alone and in mixtures. The increase in protein-UB and DNA damage was previously reported in mussels [39]. The accumulation of protein-UB tagging was also found in the mixtures but at intensities less than the combined effects from the nanoparticles alone suggesting antagonist interactions, perhaps from nCeO. The genotoxicity of reduced gold nanoparticles as with nAg was also shown in seabreams [40]. Gold nanoparticles induce DNA damage and increase nuclear abnormalities. Interestingly, the addition of gemfibrozil produced an antagonistic response as well. However, no explanation of the antagonist effect was forwarded. It is possible that the nanoparticles compete with each other to binding/sensitive sites in DNA or that internalization of nanoparticles was antagonized. For example in Figure 1, total Ag was reduced when added in mixtures while Cu and Ce were increased in tissues in mixtures. Gemfibrozil was shown to reduce lipid profiles and peroxidation which is enhanced by high glucose levels (diabetes) without affecting antioxidant enzymes superoxidase or glutathione peroxidase activities [41]. This suggests that fibrates could interact with nAg as an antioxidant/anti-inflammatory agent in a similar way to with nCeO or the redox Ce(III)/Ce(IV) couple. Increased micronuclei formation was observed in fish exposed to nAg and nCuO [42]. This was accompanied by increased malonaldehydes (LPO), catalase and superoxide dismutase. Interestingly, the 1:1 mixture of nAg and nCuO resulted in antagonist effects in DNA damage as observed in the present study with a ternary mixture of nAg, nCuO, and nCeO. However, the reason for the antagonism between nAg and nCuO remains unclear. DNA damage was also increased in the sperm of the sand dollar Scaphechinux mirabilis exposed to zinc oxide nanoparticles and zinc ions indicating that DNA damage could be produced by both the nanoparticle and dissolved zinc [43]. However, dissolved Zn did not change with the treatment groups and was not correlated with DNA damage. 

Exposure of zebrafish and THP1 cell line to nCuO and nCeO produces cytotoxic effects. However, nCuO induces gene expression in superoxide dismutase but nCeO decreased its expression [44]. When in the mixture, a low concentration (0.01 µg/mL increased more gene expression than a higher concentration (1 µg/mL). DNA damage was evaluated by the COMET assay and revealed that nCuO was more potent than nCeO and the mixture produced less (antagonism) effect (77% of the sum of DNA damage). In contrast to nCeO_2_, histopathological effects of nCuO were higher in the presence nTiO2 than these nanoparticles alone in carps [45]. The element Ti is more resistant to oxidation than Ce. In fish hepatoma cells, the effects of nZnO and nCuO mixture was enhanced and did not involve the mobilization of zinc ions [46]. The toxicity of nCuO was reduced when Zn^2+^ was added to the incubation media leading to an increase in labile Zn in cells. However, the labile Zn levels were not changed by either nCuO or nZnO suggesting that Zn ions did not interfere with the toxicity of nCuO and nZnO. In the present study, no change in labile Zn and total Zn was found suggesting that other mechanisms were at play. Damaged proteins are tagged by polyubiquitin for removal by autophagic process and nanoparticles but no the ionic counterpart was shown to specifically activate this response [39]. In fish exposed to nAg and nTiO2, exposure alone and in mixtures led to increased HSI [47] as observed in the present study as well with nCuO and nCeO alone and in both the mixtures. In the presence of nTiO2, Ag bioaccumulated less in the liver. It appears, therefore, that mixture interactions exist between nanoparticles and could involve the release of ionic metals/elements. However, the nature of these interactions is largely unknown. For example, reduced nanoparticles (nCu or nZn) are more toxic than the oxide counterpart (nCuO and nZnO) to bioluminescent bacteria [48]. Moreover, antagonist effects between nZnO and nCu toxicity could result from interactions between dissolved Cu and Zn. In this study, the ionic Cu concentration where lower in the presence of nZnO than nCu suspensions alone, which could explain the lower toxicity of nCu in the presence of nZnO. In the present study, the levels of labile Zn were not significant between treatment groups suggesting no change in Zn displacement in tissues. This suggests that the antagonist effects (reduced LPO of nCuO in mixture or decreased Ag uptake in tissues in mixtures or the effects on protein-UB and DNA damage) found in mixtures do not involve reduced mobilization of free ions from interaction with the oxide NPs. It is possible that Zn mobilization occurred within proteins or other sites without any net increase in labile Zn. However, the toxicity of nCuO to freshwater mussels did not involve any release of labile Cu in tissues [34]. However, nAg was shown to release ionic Ag^+^, which could interact with the nCuO and nCeO. If this holds true, the nAg-driven effects would differ when in the presence of nCuO and nCeO mixtures. For example, Ag tissues were lower in tissues in fish exposed to the mixtures compared to nAg alone. Another example is the decrease in aCOx activity in fish exposed to nAg alone, which was not observed in the mixtures while nCuO and nCeO alone had no effects. In another study, antagonist toxicity between CdS or ZnS nanoparticles with a metal-containing mesoporous silicon dioxide nanoparticle was associated with scavenging of metal ions in algae by a Trojan horse effect [49]. Interestingly, synergic toxic effects occurred between CdS or ZnS nanoparticles with metal-free mesoporous silicon dioxide nanoparticles suggesting the absence of metal ions involvement. 

It is noteworthy that AChE activity was significantly reduced by nCeO and nCuO and the mixtures while nAg had no effects. This suggests that these effects were mediated by the nanoparticles and not free ions. Inhibition of AChE by several nanoparticles was previously shown suggesting that this enzyme could be a marker of exposure to metallic and organic-based nanoparticles [50]. It appears that AChE inhibition mostly occurred by absorption of either the substrates or the enzyme on the nanoparticles. This is consistent with the lack of significant correlation between AChE and labile Zn and total Ag, Cu, Zn, and Ce in tissues. It was shown that a relatively high dose of nAg could cause morphological and neurological damage in zebrafish [51]. Iron oxide nanoparticles were neurotoxic in rainbow trout and decreased AChE activity in brain tissues [52]. The inhibitions were associated with increased lipid peroxidation. In the Mediterranean mussel *Mytilus galloprovincialis*, exposure to zinc-coated gold nanoparticles increased total levels of Zn in tissues and inhibited AChE as well [53]. The enzyme AChE was also inhibited by zinc-coated nanoparticles of 100 nm diameter in the gills and digestive gland of mussels but not with the 50 nm diameter one. This suggests that the size of nanoparticles could also influence AChE activity. Various metal oxide nanoparticles (nCuO and nZnO) were shown to also inhibit AChE and superoxide dismutase activity in the fish *Carassius auratus* but not with nCeO_2_ [54]. In binary and ternary mixtures with nCeO_2_, antagonist interactions were observed for AChE inhibition while nCuO and nZnO mixtures showed synergy. In the current study, AChE activity was reduced in the mixture but at intensities lower than the addition of the effects of each NP (nAg, nCeO and nCuO). Indeed, the addition of AChE fold inhibition of each nanoparticle (nAg, nCeO, nCuO) would reduce its activity by 4.3-fold while in the corresponding mixture 2, AChE dropped 3.3-fold. Based on these studies, nCuO was more toxic alone and effects are lowered in the mixtures. This was corroborated in the present study based on discriminant function activity, showing that nCuO and nCeO were more closely related to the toxic effects of the mixtures than nAg for rainbow trout juveniles.

In conclusion, this study examined the toxicity of selected nanoparticles (nAg, nCeO_2_ and nCuO) alone and in two combinations (mixtures) in an attempt to shed some light on the effects of mixtures, being a more realistic scenario in the environment. This study revealed that nCeO and nCuO produced more toxicity than metallic nAg for LPO, protein damage, genotoxicity, and reduced neural activity as determined by AChE activity. In mixtures, these effects are still observed in most cases albeit at lower (antagonism) intensities than would be expected based on the single nanoparticle exposure scenario. Moreover, these dampening effects seemed independent from the mobilization of labile Zn in tissues.

## Figures and Tables

**Table 1:** The nominal concentration of nanoparticles in the exposure media.

	Mean Diameter (nm)/Zeta Potential (mv)	Ag (µg/L) 1 h 96 h	Cu (µg/L) 1 h 96 h	Ce (µg/L) 1 h 96 h
nAg 50 μg/L	52 ± 8/−45	48 33	- -	- -
nCuO 50 μg/L	60 ± 7/−14	- -	3.5 4.2	- -
nCeO 10 μg/L	50 ± 10/−18	- -	- -	1.2 0.75
Mix 1	-	16 9	1.6 1.9	0.2 0.02
Mix 2	-	49 29	3.3 2.3	1.3 0.35
Controls (aquarium)	-	0.02 0.01	1 1.3	0.01 0.004

**Table 2:** Morphological characteristics of juvenile trout exposed to nanoparticle mixtures.

Treatment	CF	HSI	Gill Somatic Index
Control	1.02 ± 0.02	0.01 ± 0.001	0.05 ± 0.004
nCuO	1.04 ± 0.04	0.05 ± 0.002 ^ac^	0.01 ± 0.001 ^a^
5nCeO	1.03 ± 0.02	0.04 ± 0.003 ^a^	0.01 ± 0.001 ^a^
nAg	1.02 ± 0.01	0.01 ± 0.001 ^bc^	0.05 ± 0.003 ^bc^
Mixture 1 (45 µg/L total)	1.02 ± 0.02	0.05 ± 0.003 ^a^	0.01 ± 0.001 ^a^
Mixture 2 (120 µg/L total)	1.16 ± 0.08	0.04 ± 0.003 ^a^	0.01 ± 0.001 ^a^

The data represent the mean with the standard error. The Mix1 consists of 20 µg/L of nAg and nCuO and µg/L of nCeO giving a net concentration of 45 µg/L. The Mix2 consists of 50 µg/L of nAg and nCuO and 10 µg/L nCeO giving a net concentration of 110 µg/L. The letter ^a^ indicates significance relative to controls, ^b^ significance relative to Mix1 and ^c^ relative to Mix2.

## Data Availability

Data will be available upon demand.
